# Enhancement of Flame Retardancy of Colorless and Transparent Semi-Alicyclic Polyimide Film from Hydrogenated-BPDA and 4,4′-oxydianiline via the Incorporation of Phosphazene Oligomer

**DOI:** 10.3390/polym12010090

**Published:** 2020-01-03

**Authors:** Xiao Wu, Ganglan Jiang, Yan Zhang, Lin Wu, Yanjiang Jia, Yaoyao Tan, Jingang Liu, Xiumin Zhang

**Affiliations:** 1 Beijing Key Laboratory of Materials Utilization of Nonmetallic Minerals and Solid Wastes, National Laboratory of Mineral Materials, School of Materials Science and Technology, China University of Geosciences, Beijing 100083, China; 2103170022@cugb.edu.cn (X.W.); 2003180029@cugb.edu.cn (G.J.); 2103170021@cugb.edu.cn (Y.Z.); 2003190023@cugb.edu.cn (L.W.); 2003190024@cugb.edu.cn (Y.J.); 2003190022@cugb.edu.cn (Y.T.); 2 School of Electrical Engineering, Beijing Jiaotong University, Beijing 100044, China

**Keywords:** colorless polyimide film, flame retardancy, phosphazene, optical properties, thermal properties

## Abstract

Enhancement of flame retardancy of a colorless and transparent semi-alicyclic polyimide (PI) film was carried out by the incorporation of phosphazene (PPZ) flame retardant (FR). For this purpose, PI-1 matrix was first synthesized from hydrogenated 3,3′,4,4′-biphenyltetracarboxylic dianhydride (HBPDA) and 4,4′-oxydianiline (ODA). The soluble PI-1 resin was dissolved in *N*,*N*-dimethylacetamide (DMAc) to afford the PI-1 solution, which was then physically blended with PPZ FR with the loading amounts in the range of 0–25 wt.%. The PPZ FR exhibited good miscibility with the PI-1 matrix when its proportion was lower than 10 wt.% in the composite films. PI-3 composite film with the PPZ loading of 10 wt.% showed an optical transmittance of 75% at the wavelength of 450 nm with a thickness of 50 μm. More importantly, PI-3 exhibited a flame retardancy class of UL 94 VTM-0 and reduced total heat release (THR), heat release rate (HRR), smoke production rate (SPR), and rate of smoke release (RSR) values during combustion compared with the original PI-1 film. In addition, PI-3 film had a limiting oxygen index (LOI) of 30.9%, which is much higher than that of PI-1 matrix (LOI: 20.1%). Finally, incorporation of PPZ FR decreased the thermal stability of the PI films. The 10% weight loss temperature (*T*_10%_) and the glass transition temperature (*T*_g_) of the PI-3 film were 411.6 °C and 227.4 °C, respectively, which were lower than those of the PI-1 matrix (*T*_10%_: 487.3 °C; *T*_g_: 260.6 °C)

## 1. Introduction

Colorless and transparent polyimide (CPI) films have been paid ever-increasing attention in recent years due to their excellent combined thermal, optical, and dielectric properties [1,2,3]. CPI films have been thought to be good candidates for functional components for advanced optoelectronic fabrication, such as substrates for flexible display devices [4,5,6], flexible organic solar cells [7], and flexible heater for wearable sensors [8]; encapsulants for displays or electronic chips [9]; and other high-tech applications. Various CPI films, including the wholly aromatic ones containing fluoro-containing units [10,11,12] and the wholly alicyclic or semi-alicyclic ones containing alicyclic or aliphatic segments [13,14,15], have been well designed and developed in the past decades.

Among the CPI films, the semi-alicyclic ones usually possess superior optical transparency, mechanical toughness, dielectric properties, and molecular designability to their wholly aromatic counterparts [16]. However, in the practical applications for semi-alicyclic CPI films, flame retardancy is often one of the most important requirements with respect to the reliability and safety of the devices. The class of UL 94 (Underwriters Laboratories Incorporated, Bentonville, AR, USA) VTM-0 is usually required [17]. Actually, flame retardancy issues have rarely been addressed for wholly aromatic PI films due to their intrinsically inflammable nature caused by the rigid-rod and highly conjugated aromatic rings and imide units in their molecular structures [18,19]. However, these structural features for wholly aromatic PI films greatly deteriorate their optical transparency due to the formation of intra- and intermolecular charge transfer complexes (CTCs) among the electron-donating diamine units and the electron-accepting dianhydride units [20,21,22]. Due to the electron mobility in the formation of CTCs, visible light is highly absorbed by the PI film, resulting in colors from deep brown to dark yellow. Semi-alicyclic CPI films are designed by eliminating or prohibiting the formation of CTCs via incorporation non-conjugated alicyclic moieties into the molecular skeleton of CPIs, thus exhibiting very pale colors from pale yellow to colorless. Although the coloration issue is efficiently resolved for CPI films, this is usually accompanied by side-effects, mainly consisting of decreased high-temperature dimensional stability (high coefficient of thermal expansion, high-CTE), and sacrifice of flame retardancy. The current research and developmental hot topics for practical CPI films also focus on these two issues.

As for the flame retardancy issue of CPI films, the incorporation of flame retardants (FR) into the film matrix seems to be one of the most efficient pathways considering the feasibility with respect to technology and cost. However, the suitable FR for CPI films should be elaborately selected in view of the special requirements of colorless and transparent polymer films. First, the FR should not deteriorate the optical transparency of the CPI film matrix; therefore, those that could form nano-scale, preferably molecular-scale combinations with the CPI matrix are preferred. Secondly, the addition of FR cannot cause the yellowing of CPI film matrix, especially at high temperature; thus, those with good thermal stability and high-temperature oxidative resistance are preferred. Finally, the selected FR have to be compatible with the European Union (EU) RoHS (Restriction of Hazardous Substances) and WEEE (Waste Electrical and Electronic Equipment) directives; thus, those containing bromine and other harmful substances cannot be used [23]. Considering the requirements of FRs for CPI films mentioned above, the useable FRs are actually very limited.

In the current work, as one of our continuous series of works developing high-performance CPI films for advanced optical applications, the enhancement of flame retardancy of one representative semi- alicyclic CPI film derived from hydrogenated 3,3′,4,4′-biphenyltetracarboxylic dianhydride (HBPDA) and 4,4′-oxydianiline (ODA) was performed by incorporation of an organic phosphazene (PPZ) oligomer FR. The effects of the addition of PPZ on the optical and thermal properties of the derived PI composite films were investigated in detail.

## 2. Materials and Methods

### 2.1. Materials

Hydrogenated 3,3′,4,4′-biphenyltetracarboxylic dianhydride (HBPDA) was synthesized in our laboratory according to our previous work [9], or it can be purchased from Xinyuankesheng Co. Ltd. (Shandong, China) and dried at 150 °C in vacuo for 24 h prior to use. 4,4′-Oxydianiline (ODA) was purchased from Wakayama Co. Ltd. (Osaka, Japan) and used as received. Phenoxyphosphazene (PPZ) oligomer was purchased from Fushimi Pharmaceutical Co. Ltd., Japan with the trademark of Rabitle^®^ FP-100 (Appearance: white solid; Melting point: 110 °C; 5% weight loss temperature: 343 °C; Phosphorus content: 13.4%; nitrogen content: 6.0%). *N*,*N*-dimethylacetamide (DMAc), *N*,*N*-dimethylformamide (DMF), γ-butyrolactone (GBL) and other solvents were obtained from Tokyo Chemical Industry Co., Ltd. (Tokyo, Japan) and purified by distillation prior to use.

### 2.2. Characterization Methods

Fourier transform infrared (FT-IR) spectra were obtained with a Tensor 27 Fourier transform spectrometer (Bruker, Ettlingen, Germany). Ultraviolet-visible (UV-Vis) spectra were recorded on a Hitachi U-3210 spectrophotometer (Tokyo, Japan) at room temperature. Prior to testing, PI samples (thickness: 50 μm) were dried at 100 °C for 1 h to remove the absorbed moisture. Yellow index (YI) values of the PI fabrics or films were measured using an X-rite color i7 spectrophotometer (Michigan, MI, USA) with PI samples at a thickness of 50 μm in accordance with the procedure described in ASTM D1925 “Test method for yellowness index of plastics”. The color parameters were calculated according to a CIE Lab equation. L* is the lightness, where 100 means white and 0 implies black. A positive a* means a red color, and a negative one indicates a green color. A positive b* means a yellow color, and a negative one indicates a blue color. Thermogravimetric analysis (TGA) and differential scanning calorimetry (DSC) were recorded on a TA-Q series thermal analysis system (New Castle, DE, USA) at a heating rate of 10 °C/min in nitrogen. Wide-angle X-ray diffraction (XRD) was conducted on a Rigaku D/max-2500 X-ray diffractometer (Tokyo, Japan) with Cu-Kα1 radiation, operated at 40 kV and 200 mA. X-ray photoelectron spectroscopy (XPS) data were obtained with an ESCALab220i-XL electron spectrometer (Thermo Fisher Scientific, MA, USA) using 300 W of MgKα radiation. The base pressure was 3 × 10^−9^ mbar. The binding energies were referenced to the C1s line at 284.8 eV from the adventitious carbon.

The limiting oxygen index (LOI) tests were carried out according to the standard of GB/T 2406.2-2009 or ISO 4589-2: 2017 (Plastics—Determination of burning behavior by oxygen index—Part 2: Ambient-temperature test). The test specimens were 160 mm ± 5 mm in length, 20.0 mm ± 0.3 mm in width, and the thickness was 20 μm ± 1 μm. The PI samples were pretreated at 23 °C under a relative humidity of 50% for 120 h in a conditioning chamber. The temperature in the laboratory during the measurement was 23 °C and relative humidity was 35%. Each of the samples was flamed with a gas burner for no longer than 10 s. The gas flow was set to 10 L/min. For each composition, 10 samples were used in one measurement series. The UL 94 VTM (Underwriters Laboratories Incorporated, Vertical Thin Material) tests were carried out according to the standard of UL 94 or ISO 9773: 1999 (Plastics—Determination of burning behavior of thin flexible vertical specimens in contact with a small-flame ignition source). The test specimens were 125 mm ± 2 mm in length, 30.0 mm ± 0.3 mm in width, and the thickness was 20 μm ± 1 μm. The PI samples were pretreated at 23 °C under a relative humidity of 50% for 48 h in a conditioning chamber and then aged at 70 °C in a hot air oven for 168 h. Each of the samples was flamed with a gas burner twice for 3 s with the flame height of 20 mm. The second flame application time began as soon as the first burning time ended. For each composition, 5 samples were used in one measurement series. The burning time of each individual test specimen, including after first (*t*_1_) and second (*t*_2_) flame applications, were recorded. The forced-flaming behavior of the PI composite films was investigated using a FTT0007 cone calorimeter (Fire Testing Technology, West Sussex, UK) according to the standard of ASTM E1354-2015. The size of the PI films was 100 mm × 100 mm × 0.05 mm (length × width × thickness). The PI samples were wrapped in an aluminum foil and exposed horizontally to 50 kW/m^2^ external heat flux. The total heat release (THR), heat release rate (HRR), smoke production rate (SPR), rate of smoke release (RSR), and carbon monoxide production (COP) values of the PI samples were recorded. The micro-morphologies of the combusted PI films were investigated using a Technex Lab Tiny-SEM 1540 field emission scanning electron microscopy (SEM) (Tokyo, Japan) with an accelerating voltage of 15 KV for imaging. Pt/Pd was spattered on each film in advance of the SEM measurements.

### 2.3. PI Synthesis and Film Preparation

The PI (HBPDA-ODA) matrix (PI-1) was synthesized according to our previously reported procedure [9]. The white fibrous resin was dried at 80 °C in vacuo for 24 h. ^1^H-NMR (DMSO-*d*_6_, ppm): 7.34–7.31 (*d*, 2H), 7.16–7.13 (*d*, 4H), 3.20–2.97 (*m*, 2H), 2.95–2.78 (*m*, 2H), 2.17–1.96 (*m*, 4H), 1.63–1.59 (*m*, 4H), 1.32–1.14 (*m*, 4H), and 1.03–0.96 (*m*, 2H).

PI-1 film was cast from the PI (HBPDA-ODA) solution by dissolving of the resin (10 g) into newly distilled DMAc (30 g) at a solid content of 25 wt.%. PI-1 film with thicknesses ranging from 10–100 μm was obtained by thermally baking the PI solution on glass substrate in flowing nitrogen (100 mL/min) according to the following continuous heating procedure: 80 °C/2 h→150 °C/1 h→180 °C/1 h→200 °C/1 h→250 °C/1 h→280 °C/1 h.

The PI/PPZ composite films were made by illustration of the PI-2 (HBPDA-ODA-PPZ-5) sample. PI-1 resin (10 g) was dissolved into DMAc (30 g) to afford a homogeneous and clear solution. The PI-1 solution was added into a 100 mL three-necked flask and then, the solution of PPZ (0.5263 g) dissolved in DMAc (1.5789 g) was added and the mixture was stirred at room temperature for 24 h to afford a viscous and homogeneous PI-2 solution with a solid content of 25 wt.%. Then, PI-2 film was prepared with a similar procedure with the PI-1 mentioned above.

The other PI composite films, including PI-3 (HBPDA-ODA-PPZ-10), PI-4 (HBPDA-ODA-PPZ-15), PI-5 (HBPDA-ODA-PPZ-20), and PI-6 (HBPDA-ODA-PPZ-25), were prepared according to a similar procedure as mentioned above.

## 3. Results and Discussion

### 3.1. PI synthesis and Film Preparation

**Figure 1:**
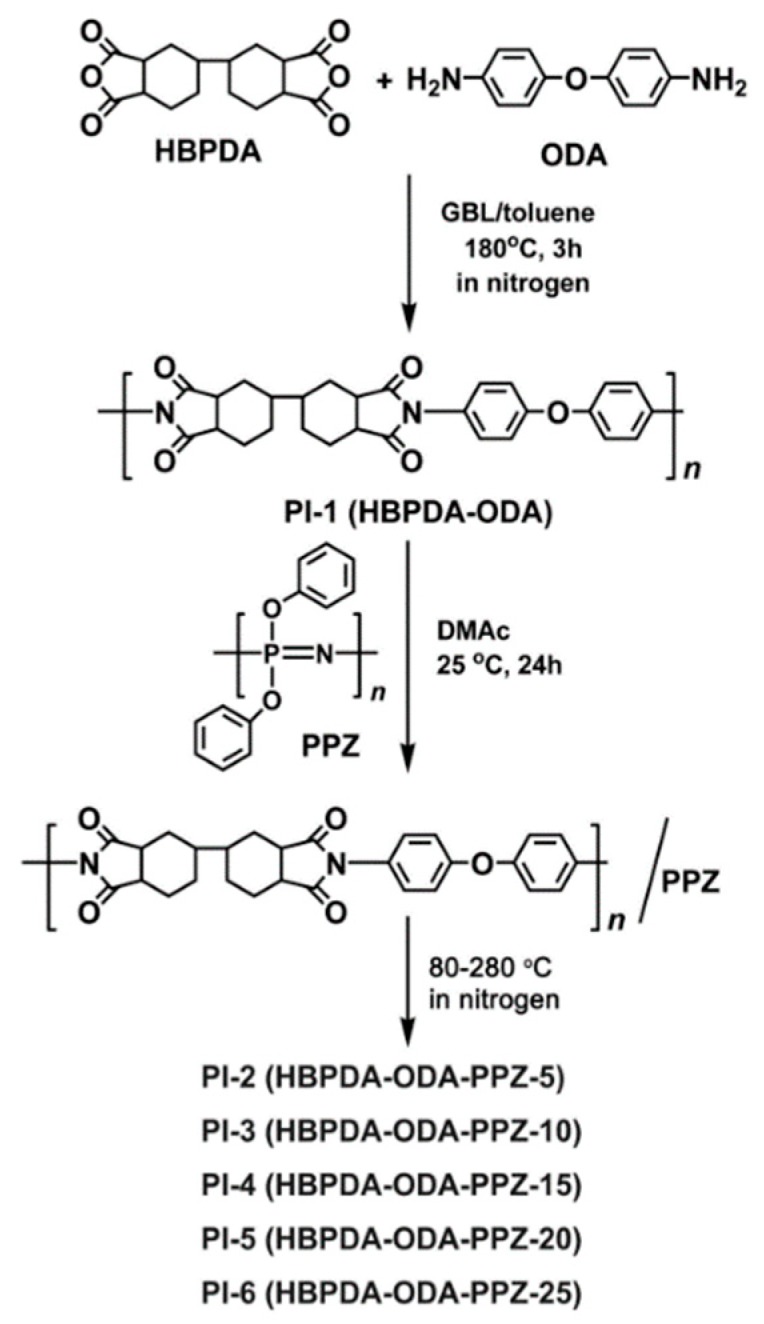
Preparation of PI-1 (HBPDA-ODA) and PI/PPZ composite films (PI-2~PI-6).

One pure PI matrix, PI-,1 and five PI/PPZ composite films, PI-2 to PI-6, with different PPZ loadings were prepared, respectively, as shown in Figure 1. PI-1 was first synthesized via the one-step high-temperature polycondensation procedure of HBPDA and ODA monomers with GBL as the solvent and toluene as the azeotropic agent. The derived PI-1 resin was easily soluble in polar aprotic solvents, such as NMP, DMF, and DMAc. DMF and DMAc have been widely used in the industrial production of PI films. The two solvents have been proven to toxic to experimental animals and humans [24]. Some less toxic solvents, such as cyclopentanone and cyclohexanone, could also be used for the current PI resins, although the solubility of the resins in such solvents might be inferior to that in the polar DMF and DMAc. PI-1 film cast from the resin solution in DMAc exhibited good film formability, optical transparency, and thermal stability. However, the film was combustible, which greatly limited its wide applications in high-tech areas. PPZ, as a class of halogen-free and highly efficient FR [25,26], has been widely used in the flame retardation improvement of polymeric textiles [27,28], coatings [29], and foams [30]. In addition, as an organic-inorganic hybrid FR [31], PPZ can usually improve the thermal stability of the common polymer matrix due to the high thermal stability of the PPZ FR. However, to the best of our knowledge, few reports have been reported up to know on the flame retardancy improvement of PI films via the incorporation of PPZ FR. In the current research, the commercially available phenoxyphosphazene oligomer was physically blended with the PI (HBPDA-ODA) matrix and a series of composite films were prepared.

**Figure 2:**
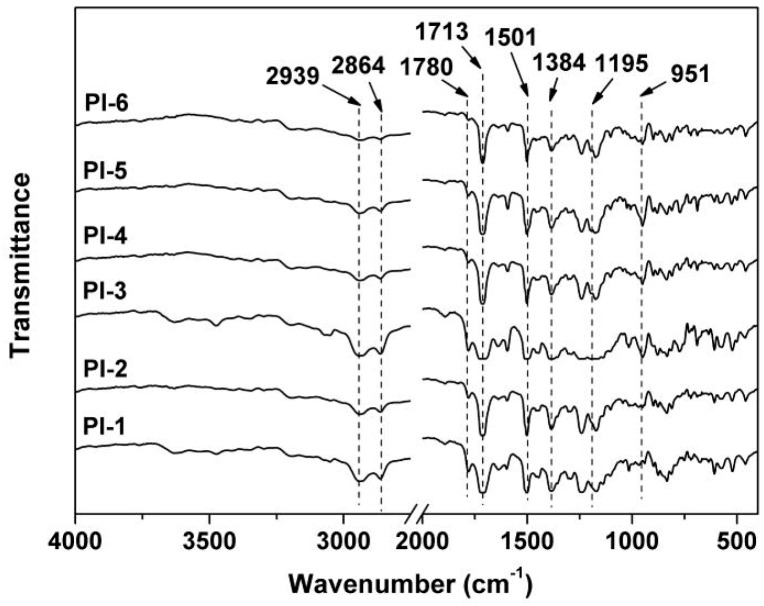
FT-IR spectra of PI films.

Figure 2 depicts the FT-IR spectra of the PI films. First, the characteristic absorptions of imide units, including the ones at 1780 cm^−1^ assigned to the asymmetrical carbonyl stretching vibrations, the ones at 1713 cm^−1^ assigned to the symmetrical carbonyl stretching vibrations, and those at 1384 cm^−1^ for the C–N stretching vibrations, were all observed. Meanwhile, the absorptions of saturated C–H stretching vibrations at 2939 and 2864 cm^−1^ of cyclohexane units in HBPDA moiety and the C=C stretching vibrations of benzene rings at 1501 cm^−1^ in ODA moiety were all detected. However, the characteristic absorptions of P–O–C bonds at 951 cm^−1^ [32] and P=N bonds at 1195 cm^−1^ [33] could only be observed in the spectra of PI-2 to PI-6, indicating the successful incorporation of the PPZ into the PI matrix. The strength of the absorption peak at 951 cm^−1^ incrementally increased in the spectra of the films and began to be clearly observed from PI-3. This indicates that the PPZ FR might form aggregates in the PI composite films when the loading ratio reached 10 wt.% and might affect the optical properties of the films, which will be discussed in detail below.

**Figure 3:**
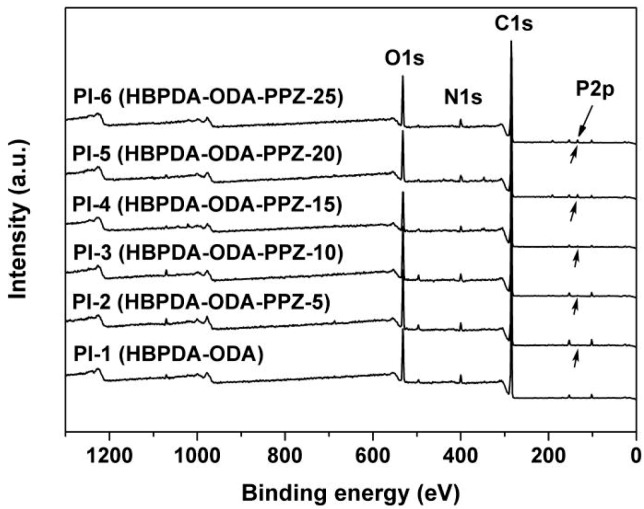
XPS spectra of PI films.

The successful incorporation of PPZ into the PI-1 matrix could further be proven by the XPS measurements, as shown in Figure 3. With the increase of PPZ loading in the composite films, the characteristic absorption of P2p began to appear, the intensity of which increased incrementally. The absorptions of common elements, including the C1s, N1s and O1s maintained in the spectra of all polymer films.

**Figure 4:**
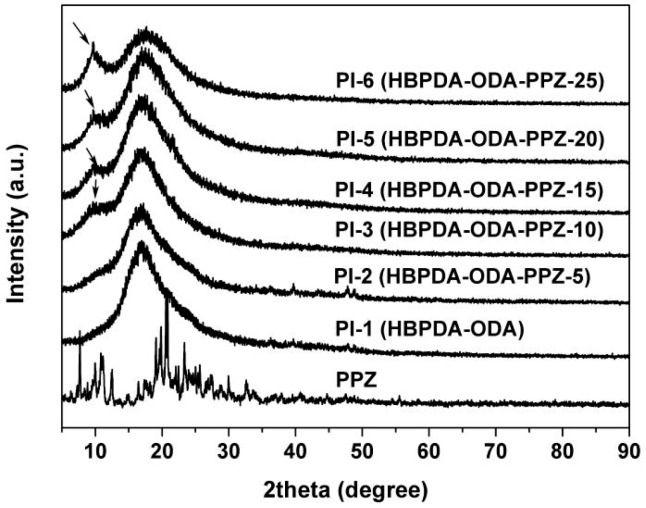
XRD spectra of PPZ (FP-100) and PI films.

Although the PPZ FR were successfully incorporated into the PI-1 matrix according to the FT-IR and XPS measurements, the miscibility of the matrix and the filler should be investigated in detail in order to justify the determine the effects of PPZ FR on the optical properties of the composite films. Figure 4 shows the wide-angle XRD spectra of PI films together with the PPZ FR. It can be clearly seen that PI-1 (HBPDA-ODA) matrix film was a typical amorphous polymer, while the PPZ oligomer possessed crystalline molecular structure with the absorption peaks in the range of 8–35°. This crystalline structure of PPZ disappeared when being combined with the PI-1 matrix at a loading content lower than 10 wt.%. PI-2 and PI-3 basically presented the nature of amorphous polymers. However, with the increase of the contents of PPZ in the composite films, some crystalline regions appear in the films. The crystallinity of the PI composite films increased with the ratio of PPZ FR. It can be predicted that the deterioration of the miscibility will undoubtedly have a significant effect on the optical properties of the PI composite films.

### 3.2. Optical Properties

The effects of the incorporation of PPZ FR on the optical properties of the composite films were investigated. In contrast to the conventional inorganic FR, PPZ is soluble in good solvent (DMAc) for the PI matrix; thus, a molecular-level combination could be anticipated. Nevertheless, the micro-phase separation between the matrix and the FR caused by the excessive loading amount or the local micro-crystallization of the PPZ FR in the composite film will inevitably affect the optical properties of the composite film.

In the current systems, the micro-phase separation and micro-crystallization of PPZ FR in the composite films with high loading amounts (PI-4, PI-5, and PI-6) was demonstrated by the XRD measurements. Thus, the optical transparency and color parameters might deteriorate to some extent. One of the main purposes of the work is to develop CPI films with good flame retardancy while maintaining intrinsic optical transparency and low yellowness and haze. Therefore, a compromise solution between the optical properties and the flame retardancy of the composite films should be achieved.

**Figure 5:**
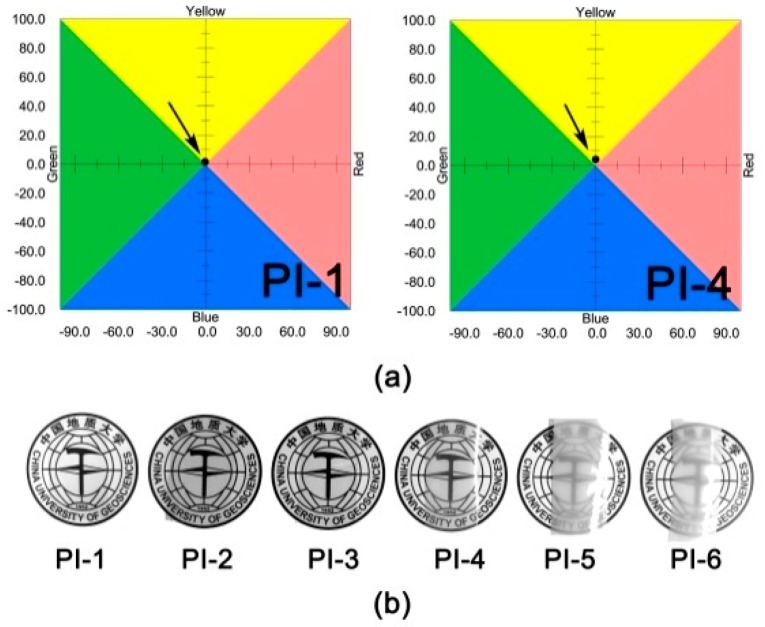
Optical properties of PI films. (**a**) CIE Lab color parameters of PI-1 and PI-4; (**b**) apparent transparency of PI films (thickness: ~50 μm).

The color parameters of the PI films were tested, and the CIE Lab values of representative PI-1 and PI-4 films, together with the apparent transparency of the PI films, are shown in Figure 5. The optical data of the PI films are summarized in Table 1. It can be seen from these optical parameters that with the increase of the content of PPZ FR in the film, the brightness (L*) of the film decreased gradually from 96.11 (PI-1) to 86.98 (PI-6), the value of a* changed from negative to positive, and the value of b* increased slightly. The results indicated that the coloration of the PI composite films showed a trend of changing from white to black and from green to red with the addition of PPZ FR. More importantly, when the content of PPZ FR in the composite film was higher than 10%, the haze values of the composite films increased sharply. This can also be confirmed by the apparent transparency of the films shown in Figure 5b. The haze value of the PI-6 film is 100%, and the film is almost opaque.

**Figure 6:**
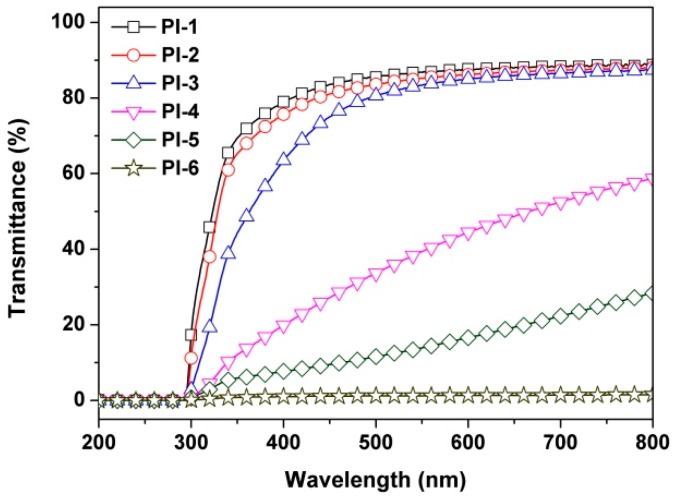
UV-Vis spectra of PI films. (film thickness: ~50 μm).

To further investigate the effects of overloading PPZ FR on the optical transparency of the PI composite films, the UV-Vis spectra of the corresponding PI films at a thickness of 50 μm were measured, and are shown in Figure 6. The cutoff wavelength (*λ*) and transmittance at the wavelength of 450 nm (*T*_450_) are tabulated in Table 1. The PI-1 matrix film showed good optical transparency in the ultraviolet–visible light region, with a *λ* value of 291 nm and a *T*_450_ value of 83.6%. The *T*_450_ values of the PI composite films decreased in the order of PI-2 (81.1%) > PI-3 (75.0%) >> PI-4 (27.2%) > PI-5 (9.5) > PI-6 (1.1%), indicating that incorporation of PPZ FR deteriorated the optical transparency of the composite films, especially at large loading amounts. This trend is basically in accordance with the haze values of the PI films, shown in Figure 5.

### 3.3. Thermal Properties

Wholly aromatic PI films are well-known for their excellent thermal resistance, which ranks at the top of polymer materials. With the introduction of alicyclic segments, the thermal stability of PI films decreases to a certain extent, but is still higher than that of ordinary optical polymer films. PPZ is also known for its good thermal stability. Thus, incorporation of PPZ FR into the PI films might maintain good thermal resistance to a great extent. The thermal stabilities of the PI films were investigated by TGA and DSC measurements, respectively. The thermal data, including temperatures at 10 wt.% weight loss (*T*_10%_), temperatures at the maximum weight loss rate (*T*_max_), and residual weight ratio at 700 °C (*R*_w700_) of the PPZ FR and the PI films are summarized in Table 2.

**Figure 7:**
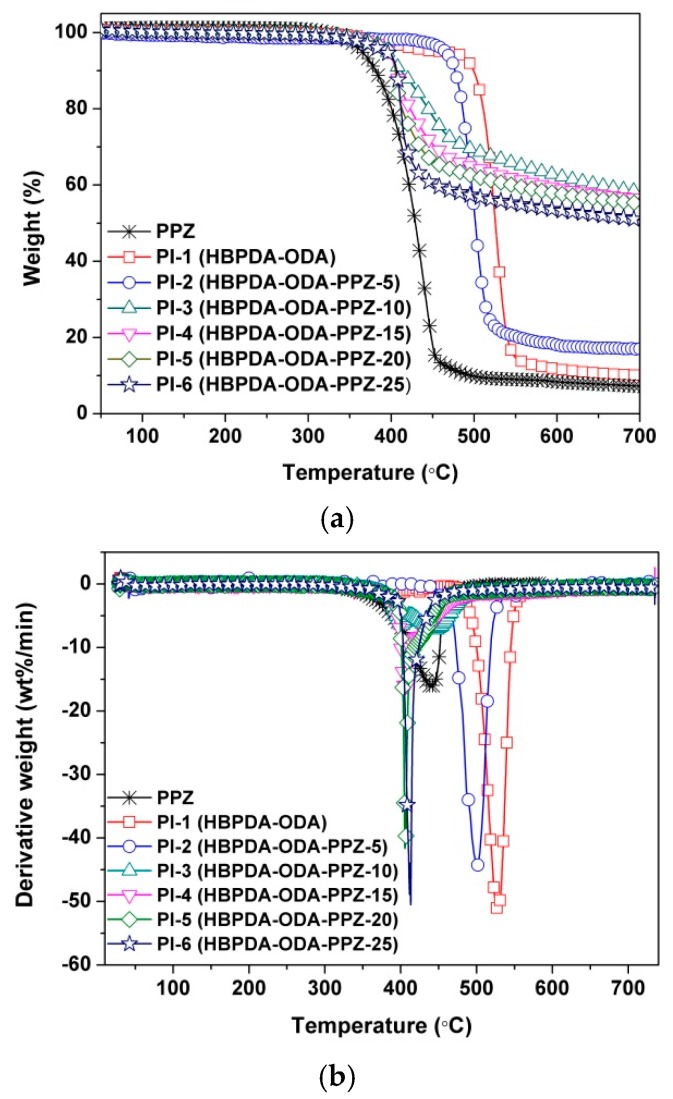
Thermal decomposition behaviors of PI films in nitrogen. (**a**) TGA; (**b**) DTG.

Figure 7 shows the TGA and the derivative TGA curves of the PPZ FR, PI-1 matrix and the PI composite films in the temperature range of 50 to 700 °C in nitrogen. It can be seen that the PPZ FR showed good thermal stability up to 350 °C, which is of high importance for the flame retardancy improvement for the PI films. On the other hand, the thermal stability of PPZ FR is inferior to that of the PI-1 matrix. The *T*_10%_ value of the PPZ was 381.3 °C, which is 106.0 °C lower than that of PI-1 film (*T*_10%_: 487.3 °C). The *T*_10%_ values of the composite films are in the range of these two limitations (381.3–487.3 °C). It can be observed that the TGA graph drastically changes between PI-2 and PI-3. This is mainly due to the inferior thermal stability of PPZ FR. The effects of the PPZ on thermal decomposition behavior are closely related with the loading ratio of PPZ and the degree of miscibility between the PPZ and the PI matrix. For PI-2 with the less PPZ loading of 5%, the effect of PPZ on the thermal stability of the composite films was not so obvious due to the good miscibility between the PPZ and the PI matrix. However, for PI-3 with a PPZ loading of 10%, this effect dramatically increased. This is, on one hand, because of the high loading of thermally unstable PPZ; on the other hand, it is due to the deterioration of miscibility. Thus, PI-3 showed a much lower thermal decomposition temperature than that of PI-2. With the further increase of PPZ, the miscibility between the PPZ and the PI matrix further deteriorated; the PI composite films showed a stable decrease in the thermal decomposition behaviors. The effects of the miscibility on the thermal decomposition behaviors of the PI composite films could also be reflected by the DTG plots shown in Figure 7b. The *T*_max_ values of the PI films and PPZ FR decreased with the order of PI-1 > PI-2 > PI-3 > PPZ > PI-4 > PI-5 ≈ PI-6. It is clear that the thermal stability of the PI composite films apparently deteriorated when the PPZ loading was higher than 10 wt.% (PI-4~PI-6).

Although the incorporation of PPZ FR somewhat decreased the initial thermal decomposition temperatures of the composite films, the char yields or residual weight ratio of the composite films at 700 °C (*R*_w700_) greatly increased. For example, PI-3 showed an *R*_w700_ value of 58.3%, which is much higher than that of the PI-1 matrix (10.0%). The *R*_w700_ values of the composite films are all higher than 50%, except for PI-2. Interestingly, the PPZ FR itself does not show a high char yield at elevated temperatures. On the contrary, it leaves less than 10% of the original weight at 700 °C. Therefore, the significant increase in char yields of the PI composite films should be due to the interaction between PPZ FR and the PI-1 matrix. This will be discussed below.

**Figure 8:**
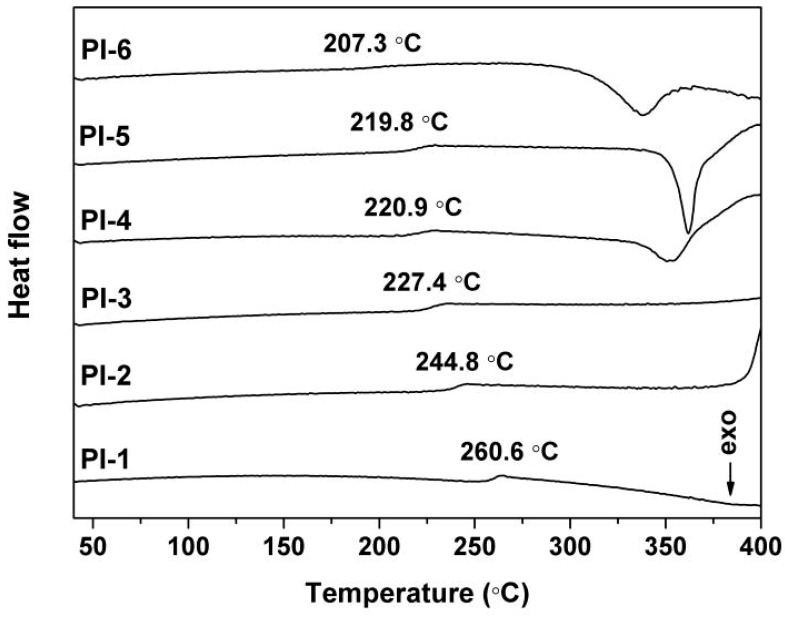
DSC plots of PI films.

The glass transition temperatures (*T*_g_) of the PI films were determined by the DSC measurements shown in Figure 8. The pristine PI-1 film showed a clear glass transition at around 260.6 °C. However, with the increase of PPZ FR contents in the composite films, the glass transition of the composite films tended to occur at lower temperature. For example, the *T*_g_ value of PI-6 was 207.3 °C, which was about 53.3 °C lower than that of PI-1. This phenomenon is mainly due to the relatively low molecular weight of the PPZ oligomer and the flexible phenoxy groups in their molecular structures. Actually, PPZs played the role of plasticizers in the composite films, inducing the glass transition of the PI composite films at relatively low temperatures. It can be clearly observed that an apparent change occurred between PI-3 and PI-4 in the DSC plots. Obvious exothermic peaks were clearly detected at around 350 °C for PI-4, PI-5, and PI-6. This indicates that the effects of the PPZ on the glass transition behaviors of the composite films are related to the loading ratio of PPZ and the degree of PPZ-PI miscibility, just like the effects on the thermal decomposition of the films. For PI-2 and PI-3 with the less PPZ loading, the effect of PPZ on the glass transitions of the polymers was not obvious due to the good miscibility between the PPZ and the PI matrix. However, for PI-4~PI-6 with higher PPZ loading, this effect appeared. This can mainly be attributed to the thermal decomposition of aggregated PPZ FR at elevated temperatures. On the other hand, the deterioration of miscibility also contributes to the change of the DSC plots.

In summary, although the thermal stability of the current PI composite films is lower than that of the PI matrix, the overall thermal stability is still high enough for their practical applications.

### 3.4. Combustion Properties

To investigate the flame retardancy of the PI composite films, the limiting oxygen index (LOI) was measured. The LOI value can be used to evaluate the flame retardancy of one polymer material. LOI is the minimum concentration of oxygen that will support the combustion of one polymer. Since the oxygen content in the air is about 21% by volume, one can deduce from the definition of LOI that if the LOI value of a polymer material is less than 21%, it is easy to burn in the air. On the contrary, if the value is higher than 21%, it tends to be nonflammable. That is to say, the higher the LOI value of one polymer, the higher its flame retardant grade is.

**Figure 9:**
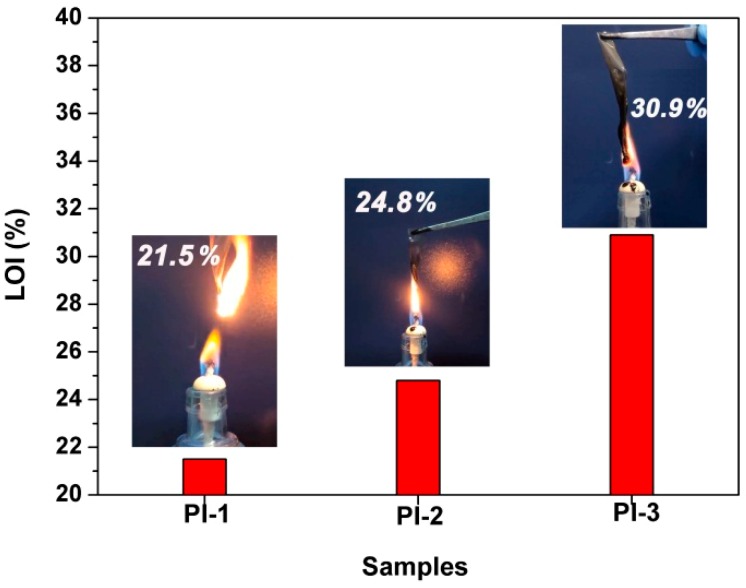
LOI values of PI films (insert: combustion of PI films).

In the current work, the flame retardancy properties of the PI composite films were evaluated by LOI, UL 94, and cone calorimetry measurements, and the combustion data are listed in Table 3. In view of the fact that PI-4~PI-6 composite films showed poor optical transparency (*T*_450_ < 75% in Table 1) due to the overload of PPZ FRs, they cannot meet the optical property requirements for practical applications. Thus, we did not measure the combustion properties of these films. According to the UL 94 measurements, the pristine PI-1 film shows a combustible nature, whereas the modified PI-2 and PI-3 show nonflammable features. As shown in Table 3, the flame retardancy class of UL 94 VTM-0 with a total combustion time (t_1_ + t_2_) lower than 20 s was achieved by introduction of PPZ FR for the PI composite films. No dripping and ignition of the cotton indicator were detected for the PI composite films in the UL 94 measurements. The LOI values of PI films are shown in Figure 9, together with the combustion conditions of PI films. Considering the deterioration of optical and thermal properties of the composite films with higher PPZ FR loadings, only PI-2 and PI-3, with the best combined properties, were tested in the flame retardancy evaluation. It can be seen from Figure 9 that the PI-1 film burned as soon as it was ignited, accompanied by sparks scattered around the ignition source. The film has a LOI value of 21.5%, which is a bit higher than that of the flammable polyethylene film (LOI: 18.0%) [34], and far lower than that of the wholly aromatic PI film, such as poly[*N*,*N*′-(oxydiphenylene)pyromellitimide] (Kapton^®^) film (LOI: 37.0%). Incorporation of PPZ FR apparently increased the LOI values of the PI films. PI-2 and PI-3 possessed LOI values of 24.8% and 30.9%, respectively, and exhibited self-extinguishing features during the combustion test. Black and tough char layers were left for both of the samples, especially for PI-3. As mentioned previously, the PI-3 film showed higher char yield at elevated temperature than those of the PI-1 matrix and the PPZ FR. The char layers undoubtedly acted as a barrier in the combustion and endowed the composite films with good flame retardancy and high LOI values. It has been well established that a thick and compact char layer is capable of effectively preventing the inner structure from being exposed to flame and releasing combustible gases [35]. To clarify the effects of residual char layers on the combustion behaviors of the films, the micro-morphology and elemental composition of the char layers after combustion test were detected.

**Figure 10:**
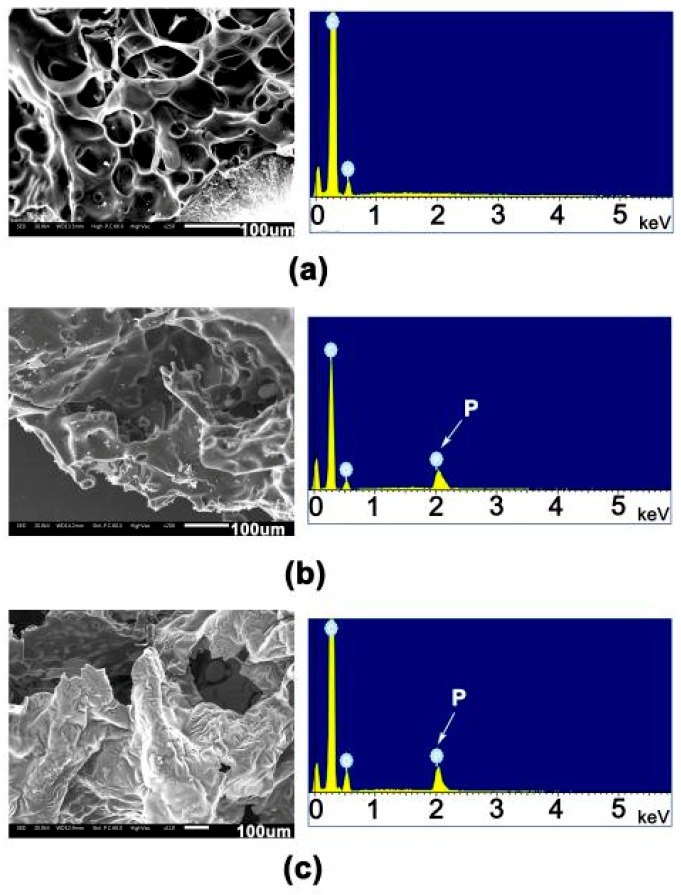
SEM images and EDX patterns of char layers in PI films after ignition. (**a**) PI-1; (**b**) PI-2; and (**c**) PI-3.

Figure 10 depicts and compares the SEM images with energy dispersive X-ray (EDX) analysis. It can be clearly observed than many round or elliptical holes can be observed in the PI-1 residues after combustion. Furthermore, the diameter of the holes near the ignition source was apparently larger than that of those far from the ignition source. EDX testing showed that the residue was mainly composed of C and O elements. For PI-2 and PI-3, more condensed char layers were observed. The results of EDX showed that phosphorus-containing passivation layers formed on the surface of the residues. This indicates that the phosphorus components might migrate to the external char layer and accumulate in condensed phase during the combustion process, which might promote to form a compact barrier to delay heat and mass transfer and thus prevent the further ignition of the films. Therefore, based on the fact that the phosphorus-containing passivation layer enriched in the surface of residues during combustion, the flame retardancy of the current PI/PPZ systems mainly matches the mechanism of condensed phase [36].

**Figure 11:**
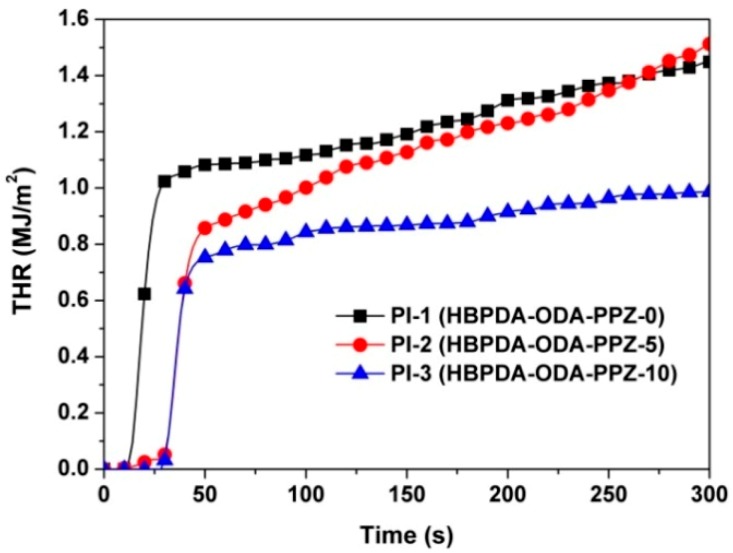
THR plots of PI films.

**Figure 12:**
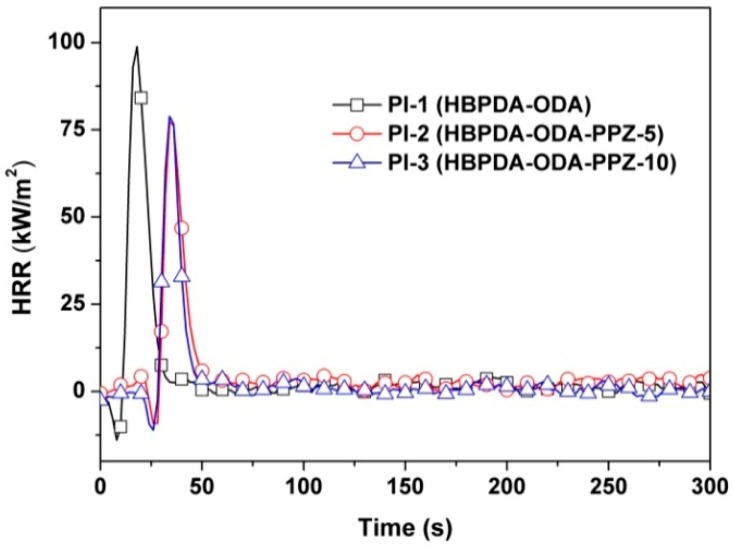
HRR plots of PI films.

The combustion behaviors of the PI films were further investigated and compared by the THR and HRR measurements, and the plots are shown in Figure 11 and Figure 12, respectively. According to the THR plots of the PI films shown in Figure 11, the THR values of the PI films decreased from 1.45 MJ/m^2^ for PI-1 to 0.99 MJ/m^2^ for PI-2, indicating the effective increase of the flame retardancy of the PI films via introduction of the PPZ FR. All of the PI films exhibited sharp peaks, with pHRR values from 76.6 to 98.9 kW/m^2^, as shown in Figure 12 and Table 3. By comparison, the PPZ-modified PI-2 and PI-3 films showed reduced pHRR values compared to those of PI-1. The combustion plots of the PI thin films agreed well with the literature [37], in which the characteristics of the current THR and HRR plots were attributed to the char-forming action during combustion. The current THR and HRR results are also in good agreement with the observation of char layers shown in Figure 10. Apparently, these inflammable and compact passivation layers provide the current PI composite films relatively good stability during the combustion test.

**Figure 13:**
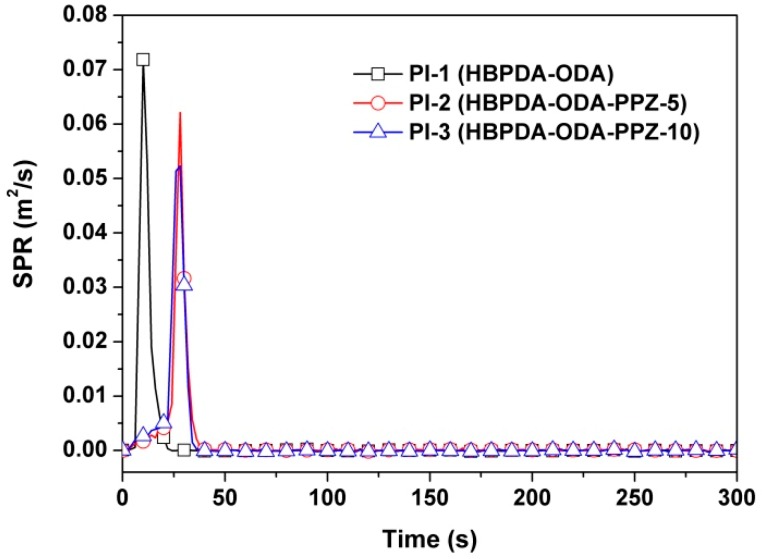
SPR plots of PI films.

**Figure 14:**
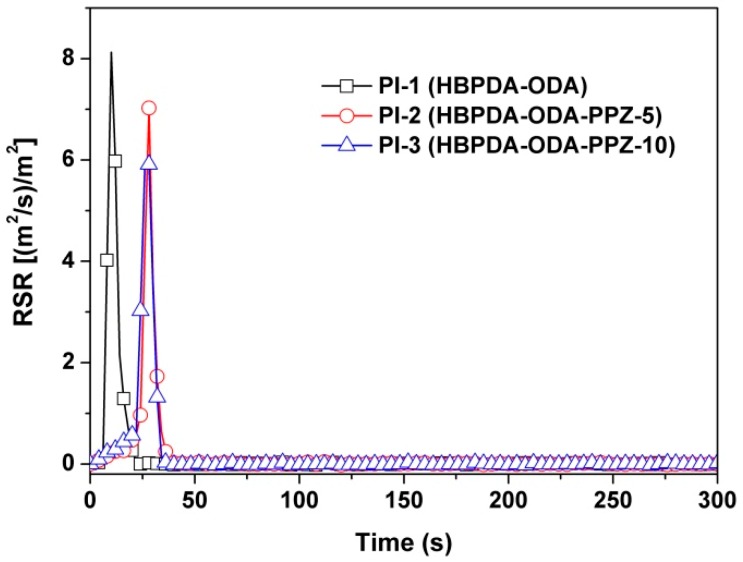
RSR plots of PI films.

**Figure 15:**
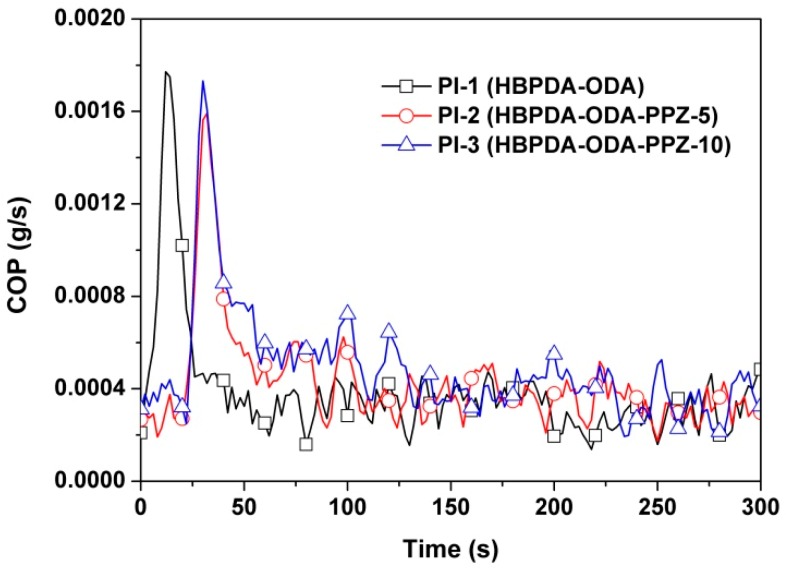
COP plots of PI films.

The smoke evaluation behaviors of the PI films were further investigated due to their high importance in possible fire scenarios. The smoke production rate (SPR) and rate of smoke release (RSR) plots of the PI films are illustrated in Figure 13 and Figure 14, respectively. It can be deduced from the figures that introduction of PPZ FR could reduce both the SPR and RSR values of the PI composite films. For instance, the peak values of PI-3 were 0.052 m^2^/s for SPR and 5.92 (m^2^/s/m^2^) for RSR, respectively, which are clearly lower than those of the pristine PI film (peak of SPR = 0.072 m^2^/s; peak of RSR = 8.13 m^2^/s/m^2^). This might be due to the good smoke suppression effects of PPZ FR [38]. Finally, the carbon monoxide production (COP) of the PI films during combustion was also investigated, and the results are shown in Figure 15. It can be seen from the figure that the introduction of PPZ FR did not obviously affect the CO release level of the PI films. However, the rate of CO production of the current PI films was much lower than that of the common polymers, such as polystyrene (PS) [39].

## 4. Conclusions

The endeavors of increasing the flame retardancy of PI (HBPDA-ODA) film while maintaining its intrinsic optical and thermal properties were successfully achieved by incorporation of PPZ oligomer FR at a loading amount below 10 wt.%. The PI-3 composite film filled with 10 wt.% PPZ FR showed the best comprehensive properties among the developed colorless and transparent PI composite films, including high flame retardancy, good thermal stability, and good optical properties. The good combined properties make the PI-3 film a good candidate for advanced optoelectronic applications.

## Figures and Tables

**Table 1:** Optical properties of PI films.

PI	PPZ (wt.%)	*λ* (nm) ^a^	*T*_450_ (%) ^b^	L* ^c^	a* ^c^	b* ^c^	Haze
PI-1	0	291	83.6	96.11	−0.23	2.57	1.21
PI-2	5	292	81.1	95.10	−0.54	4.21	3.79
PI-3	10	294	75.0	94.87	−0.27	3.56	5.03
PI-4	15	295	27.2	92.37	0.16	4.15	27.67
PI-5	20	295	9.5	87.95	0.11	3.42	93.83
PI-6	25	298	1.1	86.98	0.09	4.65	100.00

^a^*λ*: Cutoff wavelength; ^b^*T*_450_: Transmittance at the wavelength of 450 nm with a thickness of 50 um; ^c^L*, a*, b*, see 2.2. Characterization methods.

**Table 2:** Thermal properties of PPZ FR and PI films.

Samples	PPZ (wt.%)	*T*_g_ (°C) ^a^	*T*_10%_ (°C) ^b^	*T*_max_ (°C) ^b^	*R*_w700_ (%) ^c^
PPZ	100	ND ^d^	381.3	438.2	8.2
PI-1	0	260.6	487.3	527.7	10.0
PI-2	5	244.8	470.2	501.7	17.1
PI-3	10	227.4	411.6	448.9	58.3
PI-4	15	220.9	405.5	413.2	56.3
PI-5	20	219.8	405.1	407.1	55.2
PI-6	25	207.3	406.6	405.0	50.8

^a^*T*_g_: Glass transition temperature; ^b^*T*_10%_: Temperatures at 10% weight loss; ^c^*R*_w700_: Residual weight ratio at 700 °C in nitrogen; ^d^ Not detected.

**Table 3:** Combustion data of PI composite films.

PI	UL 94	t_1_ (s)	t_2_ (s)	t_1_ + t_2_ (s)	Dripping ^a^	Ignition ^b^	LOI ^c^ (%)	THR ^d^ (MJ/m^2^)	pHRR ^e^ (kW/m^2^)
PI-1	Not VTM-2	ND ^f^	ND	ND	Yes	Yes	21.5	1.45	98.9
PI-2	VTM-0	11	8	19	No	No	24.8	1.48	77.3
PI-3	VTM-0	15	0	15	No	No	30.9	0.99	76.6

^a^ If dripping in the UL 94 measurements; ^b^ If the cotton indicator was ignited in the UL 94 measurements; ^c^ limited oxygen index; ^d^ Total heat release; ^e^ Peak of heat release rate; ^f^ Not detected.

## References

[1] Ni H.J., Liu J.G., Wang Z.H., Yang S.Y. (2015). A review on colorless and optically transparent polyimide films: Chemistry, process and engineering applications. J. Ind. Eng. Chem..

[2] Zhang X.M., Song Y.Z., Liu J.G., Yang S.Y. (2016). Synthesis and properties of cost-effective light-color and highly transparent polyimide films from fluorine-containing tetralin dianhydride and aromatic diamines. J. Photopolym. Sci. Technol..

[3] Tsai C.L., Yen H.J., Liou G.S. (2016). Highly transparent polyimide hybrids for optoelectronic applications. React. Funct. Polym..

[4] Bae W.J., Kovalev M.K., Kalinina F., Kim M., Cho C.K. (2016). Towards colorless polyimide/silica hybrids for flexible substrates. Polymer.

[5] Spechler J.A., Koh T.W., Herb J.T., Rand B.P., Arnold C.B. (2015). A transparent, smooth, thermally robust, conductive polyimide for flexible electronics. Adv. Funct. Mater..

[6] Kim Y.M., Song C.H., Kwak M.G., Ju B.K., Kim J.W. (2015). Flexible touch sensor with finely patterned Ag nanowires buried at the surface of a colorless polyimide film. RSC Adv..

[7] Kang S.B., Kim H.J., Noh Y.J., Na S.I., Kim H.K. (2015). Face-to-face transferred multicrystalline ITO films on colorless polyimide substrates for flexible organic solar cells. Nano Energy.

[8] Choi S.J., Kim S.J., Jang J.S., Lee J.H., Kim I.D. (2016). Silver nanowire embedded colorless polyimide heater for wearable chemical sensors: Improved reversible reaction kinetics of optically reduced graphene oxide. Small.

[9] Wu X., Liu J.G., Jiang G.L., Zhang Y., Guo C.Y., Zhang Y.J., Qi L., Zhang X.M. (2019). Highly transparent preimidized semi-alicyclic polyimide varnishes with low curing temperatures and desirable processing viscosities at high solid contents: Preparation and applications for LED chip passivation. J. Mater. Sci. Mater. Electron..

[10] Choi I.H., Chang J.H. (2011). Colorless polyimide nanocomposite films containing hexafluoroisopropylidene group. Polym. Adv. Technol..

[11] Kim S.D., Kim S.Y., Chung I.S. (2013). Soluble and transparent polyimides from unsymmetrical diamine containing two trifluoromethyl groups. J. Polym. Sci. Part A Polym. Chem..

[12] Tapaswi P.K., Choi M.C., Nagappan S., Ha C.S. (2015). Synthesis and characterization of highly transparent and hydrophobic fluorinated polyimides derived from perfluorodecylthio substituted diamine monomers. J. Polym. Sci. Part A Polym. Chem..

[13] Hasegawa M., Hirano D., Fujii M., Haga M., Takezawa E., Yamaguchi S., Ishikawa A., Kagayama T. (2013). Solution-processable colorless polyimides derived from hydrogenated pyromellitic dianhydride with controlled steric structure. J. Polym. Sci. Part A Polym. Chem..

[14] Hasegawa M., Horiuchi M., Kumakura K., Koyama J. (2014). Colorless polyimides with low coefficient of thermal expansion derived from alkyl-substituted cyclobutanetetracarboxylic dianhydrides. Polym. Int..

[15] Kobayashi Y., Fujiwara Y., Kitaoka T., Oishi Y., Shibasaki Y. (2016). Synthesis of highly transparent poly (amide–imide) s based on trimellitic acid and dependence of thermal properties on monomer sequence. React. Funct. Polym..

[16] Hasegawa M. (2017). Development of solution-processable, optically transparent polyimides with ultra-low linear coefficients of thermal expansion. Polymers.

[17] Zhang X.M., Liu J.G., Yang S.Y. (2016). A review on recent progress of R&D for high-temperature resistant polymer dielectrics and their applications in electrical and electronic insulation. Rev. Adv. Mater. Sci..

[18] Fan H.B., Yang R.J. (2013). Flame-retardant polyimide cross-linked with polyhedral oligomeric octa (aminophenyl) silsesquioxane. Ind. Eng. Chem. Res..

[19] Lin C.H., Chang S.L., Cheng P.W. (2011). Soluble high-*T*g polyetherimides with good flame retardancy based on an asymmetric phosphinated etherdiamine. J. Polym. Sci. Part A Polym. Chem..

[20] Ando S., Matsuura T., Sasaki S. (1997). Coloration of aromatic polyimides and electronic properties of their source materials. Polym. J..

[21] Zhang Q., Tsai C.Y., Li L.J., Liaw D.J. (2019). Colorless-to-colorful switching electrochromic polyimides with very high contrast ratio. Nat. Commun..

[22] Hasegawa M., Horie K. (2001). Photophysics, photochemistry, and optical properties of polyimides. Prog. Polym. Sci..

[23] Morgan A.B. (2019). The future of flame retardant polymers—Unmet needs and likely new approaches. Polym. Rev..

[24] Tolando R., Zanovello A., Ferrara R., Iley J.N., Manno M. (2001). Inactivation of rat liver cytochrome P450 (P450) by *N*,*N*-dimethylformamide and *N*,*N*-dimethylacetamide. Toxicol. Lett..

[25] Lu S.Y., Hamerton I. (2002). Recent developments in the chemistry of halogen-free flame retardant polymers. Prog. Polym. Sci..

[26] Velencoso M.M., Battig A., Markwart J.C., Schartel B., Wurm F.R. (2018). Molecular firefighting—How modern phosphorus chemistry can help solve the challenge of flame retardancy. Angew. Chem. Int. Ed.

[27] Mayer-Gall T., Knittel D., Gutmann J.S., Opwis K. (2015). Permanent flame retardant finishing of textiles by allyl-functionalized polyphosphazenes. ACS Appl. Mater. Interfaces.

[28] Salmeia K.A., Gaan S., Malucelli G. (2016). Recent advances for flame retardancy of textiles based on phosphorus chemistry. Polymers.

[29] Huang W.K., Chen K.J., Yeh J.T., Chen K.N. (2002). Curing and combustion properties of a PU-coating system with UV-reactive phosphazene. J. Appl. Polym. Sci..

[30] Kabisch B., Fehrenbacher U., Kroke E. (2014). Hexamethoxycyclotriphosphazene as a flame retardant for polyurethane foams. Fire Mater..

[31] Honarkar H., Rahimi A. (2007). Applications of Inorganic Polymeric Materials, III: Polyphosphazenes. Monatsh. Chem..

[32] Gleria M., Bolognesi A., Porzio W., Catellani M., Destri S., Audisio G. (1987). Grafting reactions onto poly (organophosphazenes). I. The case of poly [bis(4-isopropylphenoxy) phosphazene-g-polystyrene copolymers. Macromolecules.

[33] Lejeune N., Dez I., Jaffres P.A., Lohier J.F., Madec P.J., Santos J.S.O. (2018). Synthesis, crystal structure and thermal properties of phosphorylated cyclotriphosphazenes. Eur. J. Inorg. Chem..

[34] Zanini S., Riccavdi C., Orlandi M., Colombo C., Croccolo F. (2008). Plasma-induced graft-polymerisation of ethylene glycol methacrylate phosphate on polyethylene films. Polym. Degrad. Stab..

[35] Tang C., Yan H.X., Li M.N., Lv Q. (2018). A novel phosphorus-containing polysiloxane for fabricating high performance electronic material with excellent dielectric and thermal properties. J. Mater. Sci. Mater. Electron..

[36] Qian L.J., Ye L.J., Xu G.Z., Liu J., Guo J.Q. (2011). The non-halogen flame retardant epoxy resin based on a novel compound with phosphaphenanthrene and cyclotriphosphazene double functional groups. Polym. Degrad. Stab..

[37] Schartel B., Hull T.R. (2007). Development of fire-retarded materials-Interpretation of cone calorimeter data. Fire Mater..

[38] Qiu S., Wang X., Yu B., Feng X., Mu X., Yuen R.K.K., Hu Y. (2017). Flame-retardant-wrapped polyphosphazene nanotubes: A novel strategy for enhancing the flame retardancy and smoke toxicity suppression of epoxy resins. J. Hazard. Mater..

[39] Yan Y.W., Chen L., Jian R.K., Kong S., Wang Y.Z. (2012). Intumescence: An effect way to flame retardance and smoke suppression for polystyrene. Polym. Degrad. Stab..

